# Towards trans-diagnostic mechanisms in psychiatry: neurobehavioral profile of rats with a loss-of-function point mutation in the dopamine transporter gene

**DOI:** 10.1242/dmm.027623

**Published:** 2017-04-01

**Authors:** Valentina Vengeliene, Anton Bespalov, Martin Roßmanith, Sandra Horschitz, Stefan Berger, Ana L. Relo, Hamid R. Noori, Peggy Schneider, Thomas Enkel, Dusan Bartsch, Miriam Schneider, Berthold Behl, Anita C. Hansson, Patrick Schloss, Rainer Spanagel

**Affiliations:** 1 Institute of Psychopharmacology, Central Institute of Mental Health, Medical Faculty Mannheim, Heidelberg University, 68159 Mannheim, Germany; 2 Department of Neuroscience Research, AbbVie Deutschland GmbH & Co KG, 67061 Ludwigshafen, Germany; 3 Department of Psychiatry and Psychotherapy, Central Institute of Mental Health, Medical Faculty Mannheim, Heidelberg University, 68159 Mannheim, Germany; 4 Department of Molecular Biology, Central Institute of Mental Health, Medical Faculty Mannheim, Heidelberg University, 68159 Mannheim, Germany

**Keywords:** Molecular modeling, Rat mutagenesis, *In vivo* microdialysis, RDoC matrix, mGluR2/3 antagonist LY341495

## Abstract

The research domain criteria (RDoC) matrix has been developed to reorient psychiatric research towards measurable behavioral dimensions and underlying mechanisms. Here, we used a new genetic rat model with a loss-of-function point mutation in the dopamine transporter (DAT) gene (*Slc6a3*_N157K) to systematically study the RDoC matrix. First, we examined the impact of the *Slc6a3*_N157K mutation on monoaminergic signaling. We then performed behavioral tests representing each of the five RDoC domains: negative and positive valence systems, cognitive, social and arousal/regulatory systems. The use of RDoC may be particularly helpful for drug development. We studied the effects of a novel pharmacological approach metabotropic glutamate receptor mGluR2/3 antagonism, in DAT mutants in a comparative way with standard medications. Loss of DAT functionality in mutant rats not only elevated subcortical extracellular dopamine concentration but also altered the balance of monoaminergic transmission. DAT mutant rats showed deficits in all five RDoC domains. Thus, mutant rats failed to show conditioned fear responses, were anhedonic, were unable to learn stimulus-reward associations, showed impaired cognition and social behavior, and were hyperactive. Hyperactivity in mutant rats was reduced by amphetamine and atomoxetine, which are well-established medications to reduce hyperactivity in humans. The mGluR2/3 antagonist LY341495 also normalized hyperactivity in DAT mutant rats without affecting extracellular dopamine levels. We systematically characterized an altered dopamine system within the context of the RDoC matrix and studied mGluR2/3 antagonism as a new pharmacological strategy to treat mental disorders with underlying subcortical dopaminergic hyperactivity.

## Abstract (precis)

**Summary:** The first systematic RDoc study of a disease mechanism proposes dopamine transporter DAT mutant rats as a model for drug development, targeting a hyperdopaminergic state.

## INTRODUCTION

It has long been noticed that a number of different psychiatric diagnoses largely overlap in terms of their symptoms, their underlying molecular alterations and their genetic risk factors. Furthermore, high rates of comorbidity among different diagnostic groupings are seen, and several psychiatric disorders can be treated by the same medications. This highlights the ambiguities associated with the classification of mental disorders using DSM5 (Diagnostic and Statistical Manual of Mental Disorders, Fifth Edition) or ICD10 (10th revision of the International Statistical Classification of Diseases and Related Health Problems). Thus, contemporary psychiatry uses a syndrome-based disease classification that is not based on mechanisms and does not guide treatment (Stephan et al., 2016).

During the past few years an attempt has been made to fundamentally change the classification principles of psychiatric diagnoses. This new approach, the research domain criteria (RDoC) project developed by the National Institute of Mental Health (NIMH), aims for a re-categorization of psychiatric disorders based solely on measurable behavioral dimensions and underlying mechanisms (Insel et al., 2010; www.nimh.nih.gov/research-priorities/rdoc). The RDoC approach has also been implemented into the European Roadmap for Mental Disorders (Schumann et al., 2014) and worldwide, numerous psychiatric research institutions have started to implement the RDoC approach in their research activities (Insel and Cuthbert, 2015). Its main objective is to identify the precise nature of behavioral disturbances and provide a reliable basis for the development of optimal treatments (Insel et al., 2010). However, from a preclinical point of view, these goals will not be achieved without the appropriate animal models. Here, we describe the first systematic study within the framework of RDoC using an animal model of dopaminergic imbalance, caused by a loss of dopamine transporter (DAT) function. An imbalanced dopaminergic system is one of the underlying neurobiological pathomechanisms for several psychiatric conditions, such as schizophrenia (Laruelle et al., 1999; Weinstein et al., 2016), attention deficit hyperactivity disorder (ADHD) (Ohno, 2003), obsessive compulsive disorder (OCD) (Pauls et al., 2014), and alcoholism (Tupala et al., 2001; Hirth et al., 2016).

There are five domains in the RDoC matrix representing different aspects of emotional, cognitive, motivational and social behavior (www.nimh.nih.gov/research-priorities/rdoc/constructs/rdoc-matrix.shtml). Domains of the RDoC matrix are: (1) responses to aversive situations or context, such as fear and anxiety (negative valence); (2) responses to positive motivational situations or contexts, such as reward learning and consummatory behavior (positive valence); (3) cognitive systems; (4) social processes; and (5) energy balance and sleep (arousal/regulatory processes). The matrix also integrates knowledge of the neurobiological systems that underlie the behavioral constructs of each of these domains. Although many neurobiological systems control or modulate these behavioral constructs, functional impairments of the dopaminergic system seem to have a prominent role. Besides its crucial role in extrapyramidal motor control, the dopaminergic system also plays a key role in motivation, social processes and emotional responses (Berridge and Robinson, 1998; Schultz, 1998; Spanagel and Weiss, 1999; Klanker et al., 2013; Gunaydin et al., 2014).

In the present study, we used a new genetic rat model with a loss-of-function point mutation in the DAT gene (*Slc6a3*_N157K). DAT is the main contributor to dopamine (DA) homeostasis and impairment or loss of its functionality has a dramatic effect on dopaminergic signaling (Jones et al., 1998). Association between DAT gene polymorphisms and various mental illnesses has been found in humans (Cook et al., 1995; Samochowiec et al., 2006). Inherent DAT deficiency syndrome has also been described in humans (Ng et al., 2014, 2015). Hence, functional mutations of this transporter gene can be used, not only to model dopaminergic imbalance in order to study its phenotypic impact, but also to assess the role of DAT in pathological processes. Our first aim was to perform a series of experiments to fully characterize the impact of the *Slc6a3*_N157 point mutation on the function of the monoaminergic systems. To see whether the mutation caused major changes in the secondary structure of the DAT protein, which would consequently have affected ligand binding affinity and transporter turnover rate, we made a prediction analysis of protein-folding pathways in computer-simulated wild-type and N157K mutant DAT proteins. We examined the impact of the N157K mutation on DA uptake, ligand binding and intracellular and extracellular content of monoamines. The second aim was to systematically phenotype the rat mutants according to the RDoC matrix. Hence, behavioral tests were chosen to represent each of the RDoC matrix domains. In order to assess the impact of the N157K mutation on negative valence, we measured the response to acute and potential threats in a fear-conditioning paradigm and by means of the elevated plus-maze. Positive valence was measured as initial responsiveness to reward and reward learning in a sucrose preference test and an autoshaping paradigm. Cognitive processes were measured as working and spatial working memory using a novel object recognition test and a T-maze test. The social interaction test was used to monitor social processes. Finally, arousal and regulatory control was examined as psycho-motor vigilance in an open-field test and as 24 h circadian activity in home-cage environment.

It is important to note that DAT-knockout mice that have been used in previous studies suffer from dwarfism and low overall survival, which is presumably caused by impaired function of the anterior pituitary gland (Giros et al., 1996; Bossé et al., 1997; but see also Zhuang et al., 2001): this is a limitation for phenotyping. Here, we used a novel transgenic rat line that does not suffer from dwarfism and has normal survival rates. Moreover, rats are better suited for drug development, are more flexible learners and have a more elaborate behavioral repertoire compared with mice (Abbott, 2004), which was one additional aspect of our study.

In a recent publication, Hägele and colleagues (2015) demonstrated that during reward anticipation, activation of the ventral striatum was reduced in patients suffering from schizophrenia, alcohol dependence and major depression. These findings not only confirmed that similar neurobiological alterations can indeed be found in different psychiatric diagnoses but also highlighted the feasibility of identifying and targeting such coinciding dysfunctions when developing new treatment strategies. Development of new medications for psychiatric diagnoses is the ultimate goal for preclinical research, and it could be expected that the use of animal models based on the RDoC categorization may improve the chances of translation of preclinical findings to clinical drug development (Insel, 2012). Hence, the third aim of our study was to derive a new treatment approach using the RDoC framework. Pharmacological agents targeting dopaminergic neurotransmission have been used in the management of a range of psychiatric disorders (Beaulieu and Gainetdinov, 2011). However, targeting this system may be associated with severe side effects, tolerance development and non-responsiveness in up to 30% of the treated patients (Insel, 2012). Here, we tested whether the antagonist of the metabotropic glutamate receptor 2/3 (mGluR2/3) LY341495 (Kingston et al., 1998) could be used as an alternative and novel therapy to normalize the neurobiological and behavioral consequences of a hyperactive subcortical dopaminergic system. Psychostimulants activate the dopaminergic system and induce hyperactivity in healthy subjects but have a paradoxical, poorly understood, calming effect in hyperactive individuals (Di Chiara and Imperato, 1988; Gainetdinov et al., 1999). We therefore chose to block mGluR2/3 because, similar to amphetamine, LY341495 also increases extracellular DA levels in healthy animals (Hu et al., 1999).

## RESULTS

### Molecular characterization of a novel mutant rat model with a hyperactive subcortical dopaminergic system

In the N-ethyl-N-nitrosourea (ENU)-driven target-selected mutagenesis screen, we identified a point mutation in the *Slc6a3* coding sequence (exon 3) with a T/G transversion at nucleotide position 471. This nucleotide exchange leads to substitution of an asparagine amino acid residue by a lysine residue at position 157 (N157K) in the SLC6A3 (DAT) protein, which introduces new positive charge into the amino acid sequence.

**Fig. 1.**
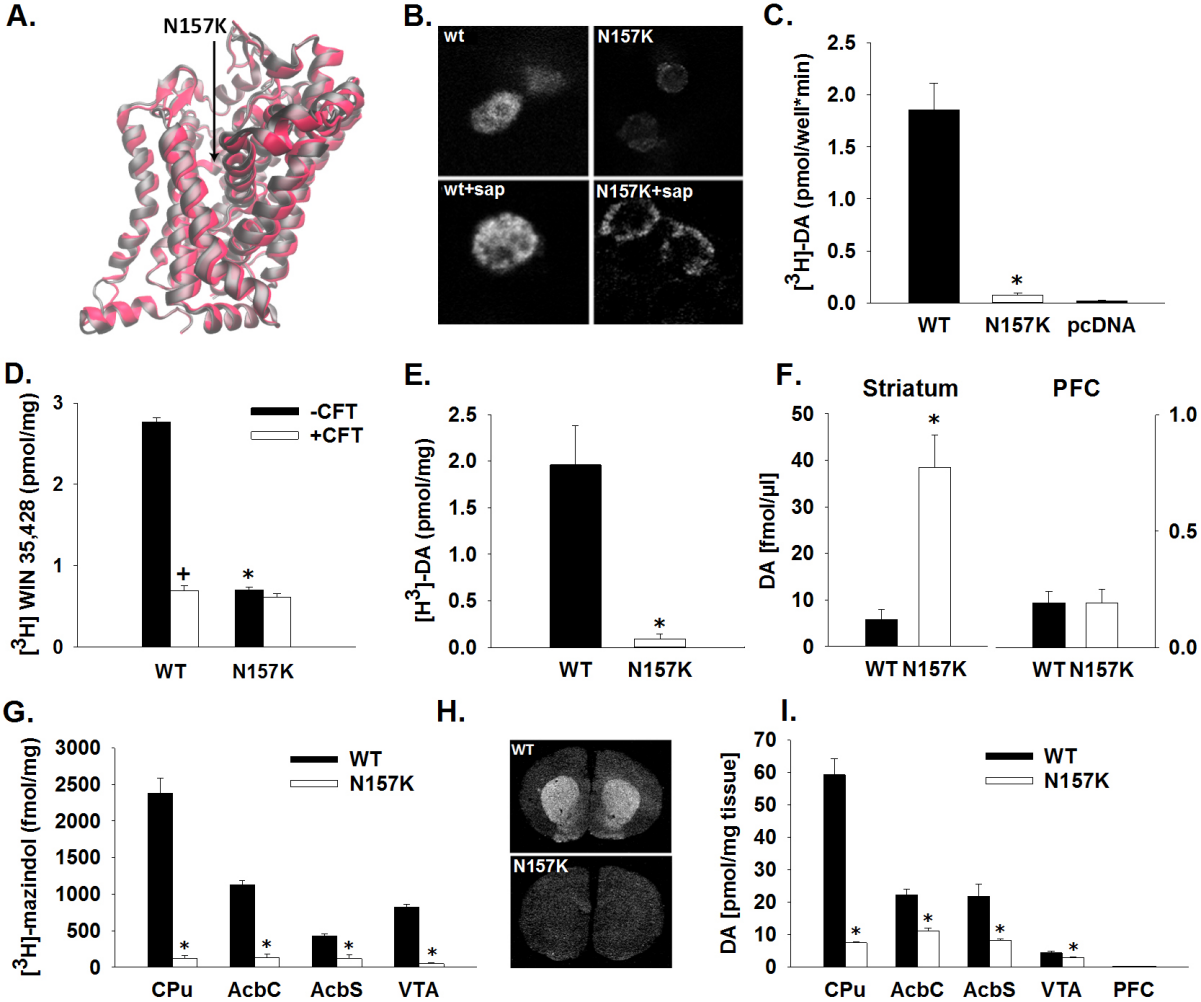
**Molecular characterization of a novel mutant rat model with a subcortical hyperdopaminergic state.** (A) Secondary structures of the wild-type (gray) and N157K-mutant (red) dopamine transporter (DAT) following energy minimization (1 ns) within an environment resembling the intracellular space. (B-D) Subconfluent HEK293 cells transiently transfected with rat DAT-wt (WT) or rat DAT-N157K (N157K). (B) Immunofluorescence and confocal analysis of DAT protein using an antibody targeted against an extracellular epitope of DAT in the absence (top row) and presence (bottom row) of detergent saponine (sap). A weak cell surface labeling is seen for DAT-N157K in the absence of detergent (top right) and a globular distribution of protein is seen for both DAT-wt and DAT-N157K (bottom row). Shown are example *z*-projections of single cells. (C) [^3^H]DA uptake in HEK293 cells expressing DAT. Specific [^3^H]DA uptake is defined as the difference between monoamine transporter mediated uptake minus control uptake in HEK293 cells that have been transiently transfected with empty vector pcDNA3.1(pcDNA). (D) Total (-CFT) and nonspecific (+CFT) binding of [^3^H]WIN35,428 to DAT. Nonspecific binding was determined in the presence of 50 µM β-CFT (WIN35,428). (E) Total [^3^H]DA uptake in synaptosomes obtained from the dorsal striatum (caudate putamen, CPu) from wild-type (WT, *n*=3) and DAT mutant (N157K, *n*=3) rats. (F) Basal extracellular DA levels in the CPu and medial prefrontal cortex (PFC) of WT (*n*=12) and N157K (*n*=9) rats. (G) Quantitative analysis of DAT ([^3^H]-mazindol) binding levels in the CPu, nucleus accumbens core (AcbC), nucleus accumbens shell (AcbS) and ventral tegmental area (VTA) of WT (*n*=10) and N157K (*n*=9) rats. (H) Representative dark-field images showing [^3^H]-mazindol binding on a coronal brain section from WT and N157K rats at the striatal level. (I) Tissue concentration of dopamine in homogenates of CPu, AcbC, AcbS, VTA and PFC of WT (*n*=5) and N157K (*n*=5) rats. All data are expressed as means±s.e.m. *P<0.05, significant difference from DAT-wt cells/WT rats; ^+^*P*<0.05, significant difference from the total binding.

This new positive charge in the amino acid sequence may lead to conformational changes of the protein structure and interfere with its affinity and efficacy or impair insertion into the plasma membrane. However, calculation of the free energies of the WT and mutant N157K DAT suggests that the mutation does not induce critical alterations in the conformation stability or any major changes in the secondary structure of DAT protein (Fig. 1A, Table S1). Thus, the root mean square deviation of the two structures [RMSD (WT, N157K)=1.6 Å] was below the resolution of the x-ray diffraction for obtaining the structure of DAT (2.95 Å). The absence of significant conformational changes is further supported by Ramachandran diagrams (Fig. S1) showing no differences in the distribution of dihedral angles of the backbones of WT and mutant DAT.

Therefore, we suggested that the mutation would impair DAT insertion into the plasma membrane. To test this, HEK293 cells were transiently transfected with either rat DAT-wt or rat DAT-N157K. In the absence of the detergent saponin, only weak immunoreactive signals could be obtained for DAT-N157K on the cell surface in contrast to an evenly distributed cell surface staining of DAT-wt transfected cells (Fig. 1B). In the presence of 0.01% saponin the antibody labeled the plasma membrane as well as punctuated signals inside the cells for both DAT-wt and DAT-N157K cells (Fig. 1B). This indicates that the rat DAT-N157K protein is transcribed and translated but is not correctly processed to the cell surface.

We then tested the functionality of DAT. Analysis of [^3^H]DA uptake into HEK293 cells expressing either DAT-wt or DAT-N157K revealed that specific [^3^H]DA uptake into the cells was significantly different between these cell lines (*t*_4_=6.9, *P*<0.01). [^3^H]DA transport in the DAT-N157K-transfected cell line was just above the detection limit of the assay (Fig. 1C).

In addition, radioligand binding revealed significant differences in total binding of [^3^H]WIN35,428 between DAT-wt and DAT-N157K transfected cell lines (*F*_3,4_=832.1, *P*<0.001). Subsequent *post hoc* analysis showed significantly different total ligand binding when compared with nonspecific binding in HEK293 cells transfected with DAT-wt (Fig. 1D). By contrast, in DAT-N157K cells, total nonspecific binding did not differ significantly, suggesting that [^3^H]WIN35,428 was unable to bind or could only bind very weakly to the target protein. We also measured [^3^H]DA uptake in the striatal synaptosomes of mutant rats and WT littermates, and found significant differences between genotypes (*t*_4_=4.4, *P*<0.05). In mutant rats, [^3^H]DA uptake was attenuated by more than 95% (Fig. 1E).

If DAT-N157K protein is not correctly processed to the cell surface and radioligand binding and DA uptake in the striatum is strongly reduced, then extracellular DA levels should be strongly increased. Indeed, by means of *in vivo* microdialysis in freely moving mutant rats, significant augmentation in basal DA content in the caudate putamen nuclei (CPu) was measured (*t*_18_=4.9, *P*<0.001) (Fig. 1F). Concentrations of DA metabolites also tended to be higher in mutant rats [3,4-dihydroxyphenylacetic acid (DOPAC): *P*=0.06; HVA: *P*=0.17]. Baseline levels of the serotonin [5-hydroxytryptamine (5-HT)] metabolite 5-hydroxyindoleacetic acid (5-HIAA), glutamate and glycine were similar in both genotypes (5-HIAA: *P*=0.23; Glu: *P*=0.39; Gly: *P*=0.56) (data not shown). DA levels in the medial prefrontal cortex (PFC) were very low at 0.19 fmol/µl and 0.19 fmol/µl, for WT and mutant rats, respectively, and not different between genotypes (*P*=0.99) (Fig. 1F).

To see if compensation ensues in the serotonergic (5-HT) and norepinephrine (NE) systems, we performed: (1) binding studies for the different monoamine transporters and (2) a neurochemical screen in *Slc6a3*_N157K mutant rats. Autoradiography data analysis revealed that [^3^H]-mazindol binding to DAT protein was significantly lower in mutant rats compared with WT in CPu (*t*_17_=10.3, *P*<0.001), nucleus accumbens core (AcbC) (*t*_17_=12.8, *P*<0.001), nucleus accumbens shell (AcbS) (*t*_17_=5.6, *P*<0.001) and ventral tegmental area (VTA) (*t*_17_=18.1, *P*<0.001) (Fig. 1G,H). In the PFC and amygdala (Amy), [^3^H]-mazindol binding was below the detection limit. Binding of [^3^H]-citalopram to the 5-HT transporter SERT was also different between genotypes. Specifically, SERT levels were increased in AcbC (*t*_16_=5.3, *P*<0.001), AcbS (*t*_16_=4.7, *P*<0.001), CPu (*t*_17_=2.7, *P*<0.05) and VTA (*t*_16_=4.4, *P*<0.001), but SERT levels in substantia nigra pars reticulata (SNr) were not affected by mutation (Fig. S2A). Data analysis of [^3^H]-nisoxetine binding assay demonstrated that levels of the norepinephrine transporter (NET) were reduced in AcbC (*t*_16_=4.1, *P*<0.001), AcbS (*t*_14_=3.7, *P*<0.01) and VTA (*t*_16_=10.0, *P*<0.001) in mutant animals. NET levels in the CPu were not affected by mutation (Fig. S2B).

In the neurochemical screen DA, 5-HT and NE content and the respective metabolites were measured in different brain sites. The tissue content of DA in CPu, AcbC, AcbS and VTA was significantly different between genotypes (CPu: *t*_8_=10.4, *P*<0.001; AcbC: *t*_8_=5.7, *P*<0.001; AcbS: *t*_8_=3.6, *P*<0.01; VTA: *t*_8_=2.2, *P*=0.05) (Fig. 1I). No changes in tissue DA levels were detected in the PFC (*P*=0.47) (Fig. 1I), substantia nigra (SN) (*P*=0.14) or Amy (*P*=0.72) (data not shown). Concentration of the DA metabolites DOPAC and HVA were similar between genotypes, with the exception of a strong increase in HVA levels in CPu (*t*_8_=9.3, *P*<0.001) and AcbC (*t*_8_=5.4, *P*<0.001) (Fig. S3A,B). Accordingly, turnover ratios DA/DOPAC and DA/HVA were significantly reduced in CPu, AcbC and AcbS, indicating that the metabolic rate of DA was increased in these brain areas (data not shown). NE levels were also different between genotypes in CPu, AcbS and VTA (CPu: *t*_8_=3.2, *P*<0.05; AcbS *t*_8_=2.5, *P*<0.05 and VTA: *t*_8_=2.7, *P*<0.05). NE content in the AcbC (*P*=0.18), PFC (*P*=0.30), SN (*P*=0.48) and Amy (*P*=0.82) were similar between genotypes (Fig. S3C). Reduced 5-HT levels were found in CPu (*t*_8_=7.5, *P*<0.001) and AcbS (*t*_8_=4.3, *P*<0.01) of mutant rats compared with WT rats. Similar levels of 5-HT between genotypes were found in the AcbC (*P*=0.15), VTA (*P*=0.14), PFC (*P*=0.22), SN (*P*=0.05) and Amy (*P*=0.74) (Fig. S3D). HIAA concentrations did not differ between genotypes (Fig. S3E) but the turnover ratio of 5-HT/5-HIAA was significantly reduced in CPu, AcbC and AcbS of mutants, suggesting that 5-HT metabolism was increased in these brain areas (data not shown).

Finally, alterations in monoamine transporter systems and accompanying extracellular mononamine levels may affect D1- and D2-like DA receptors. Analysis of [^3^H]-SCH23390 and [^3^H]-raclopride binding data revealed that the mutation caused a significant reduction in D1- and D2-like receptor binding levels in nearly all analysed brain structures (CPu: *t*_17_=14.6, *P*<0.001 and *t*_17_=5.5, *P*<0.001; AcbC: *t*_16_=14.0, *P*<0.001 and *t*_17_=3.2, *P*<0.01; AcbS: *t*_16_=16.2, *P*<0.001 and *t*_17_=2.7, *P*<0.05 for [^3^H]-SCH23390 and [^3^H]-raclopride binding, respectively) (Fig. S4A,B). D1 receptor levels were also significantly changed in VTA (*t*_12_=4.3, *P*<0.01) and the SNr (*t*_17_=7.6, *P*<0.001); however, no changes in D1-like binding were found in the PFC (data not shown).

### Systematic analysis of the DAT-N157K rat mutants according to the RDoC matrix

#### Domain: negative valence systems

**Fig. 2.**
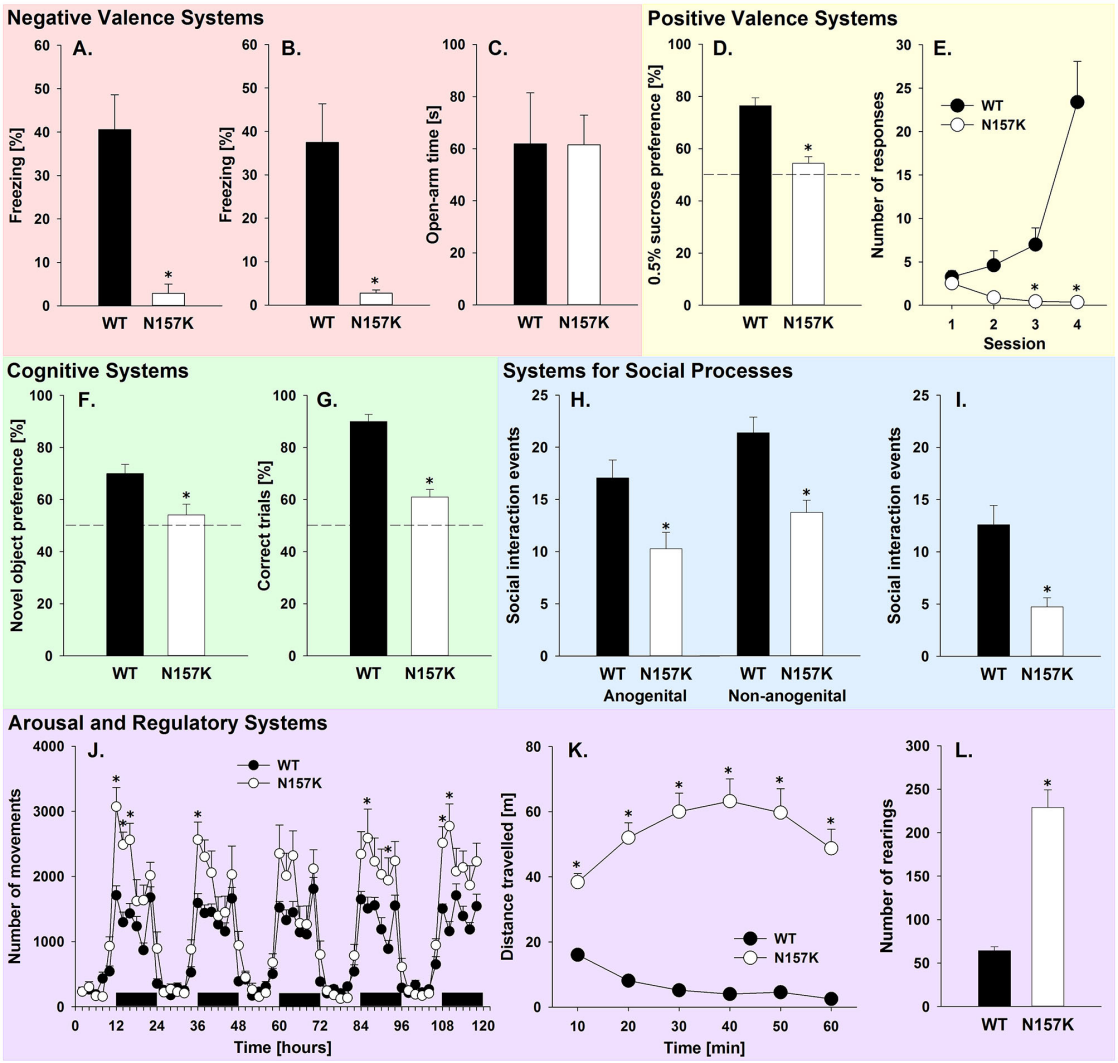
**Systematic analysis of the DAT-N157K mutant rats according to the RDoC matrix.** (A-C) Negative valence systems are represented as time spent freezing (calculated as percentage of the total exposure time) during footshock-conditioned (A) context and (B) cue in wild-type (WT, *n*=6) and DAT mutant (N157K, *n*=5) rats in fear-conditioning paradigm. (C) Time (s) spent in open arms by WT (*n*=9) and N157K (*n*=15) rats during a 5 min plus-maze test. (D,E) Positive valence systems are represented as (D) preference for 0.5% sucrose solution over water during the 24-h free-choice sucrose preference test in WT (*n*=16) and N157K (*n*=25) rats and as (E) number of conditioned responses during the autoshaping task in WT (*n*=15) and N157K (*n*=11) rats. In this task, the first three daily sessions consisted of 20 trials and the fourth session consisted of 40 trials. (F,G) Cognitive systems are represented as (F) time spent exploring a novel object (calculated as percentage of the total exploration time) during the novel object recognition test in WT (*n*=22) and N157K (*n*=23) rats and as (G) percentage of correct choices during the T-maze spontaneous alternation test in WT (*n*=5) and N157K (*n*=5) rats. (H,I) Systems for social processes are represented as number of (H) anogenital and non-anogenital exploration and (I) approach/following events in WT (*n*=12) and N157K (*n*=11) rats during 5 min interaction with an unknown social partner in the social interaction test. (J-L) Arousal and regulatory systems are represented as (J) circadian activity recordings in WT (*n*=11) and N157K (*n*=9) rats measured as the number of movements during five consecutive days in the home cage [black horizontal bars mark the dark (active) phases of the circadian cycle] and as locomotor vigilance measured as (K) distance travelled (m) every 10 min and as (L) total number of rearings in the 60 min open-field test in WT (*n*=20) and N157K (*n*=24) rats. All data are expressed as means±s.e.m. *P<0.05, significant difference from WT rats.

Negative valence systems are primarily responsible for responses to aversive stimuli or context, such as fear and anxiety. According to the RDoC matrix, DAT and DA play a role in fear but not anxiety. DAT-N157K mutants were first tested in fear conditioning. In mutant rats, freezing time was lower compared with WT during exposure to context conditioned to footshock (*t*_9_=4.2, *P*<0.01) (Fig. 2A) and during exposure to the cues associated with the footshock both before (*t*_9_=3.5, *P*<0.01) (Fig. 2B) and after (*t*_9_=2.5, *P*<0.05) (data not shown) the extinction session.

Anxiety-like behavior was tested on the elevated plus maze. No difference between WT and mutant rats in either open arm time (*P*=0.99) or number of open arm visits (*P*=0.50) was measured in this anxiety test, demonstrating unaltered anxiety levels in DAT-N157K mutant rats (Fig. 2C). The number of visits to the closed arms was not significantly different between genotypes (*P*=0.18), indicating similar activity levels during this test (data not shown).

#### Domain: positive valence systems

Positive valence systems are primarily responsible for responses to positive motivational stimuli or context, such as responsiveness to reward and reward learning. According to the RDoC matrix, DAT and DA should play a role in responsiveness to reward (assessed here using the sucrose preference test) and reward learning (assessed here with a food reward-based autoshaping learning task). Data analysis revealed that preference for 0.5% sucrose solution over water during the 24 h free-choice sucrose preference test was significantly different between genotypes (*t*_39_=5.5, *P*<0.001). Mutant animals had almost equal preference for both fluids, i.e. preference for sucrose was 54% (Fig. 2D).

Two-way ANOVA with repeated measures revealed that DAT-N157K mutant rats were different from their WT littermates in their performance during the autoshaping task (factor genotype: *F*_1,24_=13.0, *P*<0.01 and factor genotype×session interaction: *F*_3,72_=16.3, *P*<0.001) (Fig. 2E). Specifically, WT rats rapidly developed lever-pressing behavior in order to receive a food reward, whereas mutant rats failed to learn a stimulus-reward association and did not approach a reward-predicting stimulus. This difference between genotypes was already seen during the later training sessions but was especially evident in the final test session.

#### Domain: cognitive systems

Most aspects of the domain cognitive systems seem to be independent of DAT; however, DAT seems to have a great impact on working memory. To assess this in mutant rats, animals were submitted to novel object recognition and T-maze testing. Percentage object discrimination time during initial 3 min sample phase did not differ between the genotypes (*P*=0.74), showing no side preference to the experimental apparatus. However, a comparison of exploration time of the novel object revealed that genotypes were significantly different in the object recognition memory (*t*_43_=2.9, *P*<0.01) (Fig. 2F). Performance of mutant rats was clearly below that of WT littermates since the time they spent exploring the new object was 54% of total exploration time. Total exploration time did not differ between genotypes (*P*=0.23 and *P*=0.31 for the sample and discrimination phase, respectively), showing that differences in locomotor activity (see ‘Domain: arousal/regulatory systems’ section) did not significantly interfere with animal behavior during this test. Analysis of the data collected in the T-maze test revealed significant differences between genotypes in spontaneous alternation behavior (*t*_8_=7.3, *P*<0.001). Mutant rats exhibited significantly fewer correct choices (i.e. entered the unexplored arm) during the test than their WT counterparts (Fig. 2G).

#### Domain: systems for social processes

DAT is not listed in the RDoC matrix for any aspect of the domain social processes. Nevertheless, we tested DAT-N157K mutant rats in the social interaction test and found significant differences for anogenital exploration (*t*_21_=2.9, *P*<0.01), non-anogenital exploration (*t*_21_=4.0, *P*<0.001) and approach/following behavior (*t*_21_=3.8, *P*<0.01) (Fig. 2H,I), as well as significantly different total social interaction events (*t*_21_=4.3, *P*<0.001) and total exploration time (*t*_21_=2.6, *P*<0.05) (data not shown). Contact behavior, such as grooming and crawling over, was very low in WT rats and absent in mutant animals (data not shown). There was no significant difference in self-grooming (*P*=0.47) and social evasion (*P*=0.85) (data not shown).

#### Domain: arousal/regulatory systems

Arousal and circadian rhythmicity are constructs of this domain, where arousal is strongly determined by DAT and DA function, whereas circadian rhythmicity is controlled by clock genes. We assessed both home-cage activity with diurnal rhythmicity and open-field activity. In the home cage, two-way ANOVA revealed that the total number of movements measured during five consecutive days was significantly different between genotypes (*F*_1,18_=11.8, *P*<0.01). However, increased activity could be measured only during the active (dark) phase of their daily cycle, and diurnal activity remained intact in DAT-N157K mutant rats (*P*<0.01 and *P*=0.10 for average activity during active and inactive phase, respectively) (Fig. 2J). In the open field, data analysis demonstrated that DAT mutation caused significant changes in both horizontal (total distance traveled) (*t*_42_=10.0, *P*<0.001) and vertical (number of rearings) (*t*_42_=7.3, *P*<0.001) activity. Mutant rats exhibited dramatically increased total distance traveled compared with WT littermates (Fig. 2K). Similarly, during 60 min of open field testing, mutant animals had a significantly higher number of rearings when compared with WT rats (Fig. 2L).

### Pharmacological reversal of phenotypic alterations in DAT-N157K rats

**Fig. 3.**
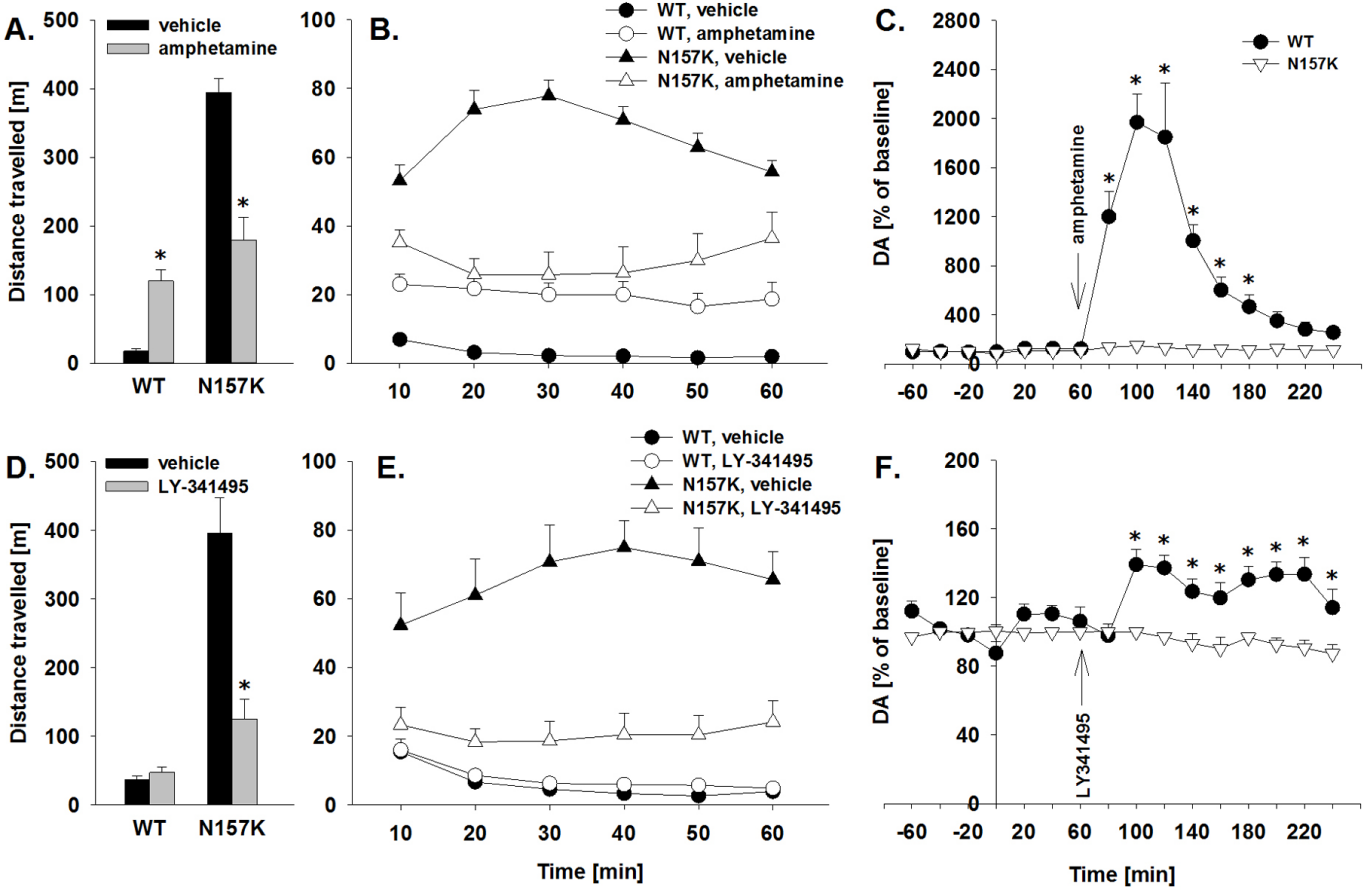
**Pharmacological reversal of phenotypic alterations in DAT-N157K rats.** (A,B,D,E) Distance traveled (cm) during 60 min testing in the open field in wild-type (WT, *n*=7-15) and DAT mutant (N157K, *n*=6-18) rats. Thirty minutes before the test, all rats were administered vehicle, 2 mg/kg amphetamine (A,B) or 10 mg/kg LY-341495 (D,E). The data are shown as total distance traveled (A,D) and as distance traveled every 10 min (B,E). (C,F) Extracellular DA levels in the caudate putamen of WT (*n*=6-7) and N157K (*n*=5-7) rats. At the time point 0 min, all rats were administered with the vehicle; 60 min later animals were given either 2 mg/kg amphetamine (C) or 10 mg/kg LY341495 (F). Microdialysis samples were collected every 20 min. All data are expressed as means±s.e.m. *P<0.05, significant difference from WT rats.

One-way ANOVA revealed that administration of all three compounds – amphetamine, atomoxetine and LY341495 – significantly and dose-dependently (note that only the highest dose is shown in the figures) reduced total distance traveled in DAT-N157K mutant rats (factor treatment group: *F*_2,33_=13.6, *P*<0.001; *F*_2,30_=16.2, *P*<0.001 and *F*_3,29_=30.1, *P*<0.001 for amphetamine, atomoxetine and LY341495, respectively) (Fig. 3; Fig. S5A,B). Administration of amphetamine significantly increased locomotor activity in WT rats (factor treatment group: *F*_2,27_=34.7, *P*<0.001). In contrast, atomoxetine treatment effects in WT rats were similar to those seen in mutant rats (factor treatment group: *F*_2,18_=6.4, *P*<0.01), suggesting nonselective reduction of motor activity by this compound (Fig. S5A,B). LY341495 treatment had no effect on the open-field activity in WT rats (*P*=0.37) (Fig. 3D,E).

Analysis of results obtained from the microdialysis in freely moving rats demonstrated that administration of amphetamine significantly increased extracellular DA levels and decreased DOPAC and HVA levels in WT animals (factor time×genotype interaction: *F*_15,165_=22.8, *P*<0.001; *F*_15,165_=5.6, *P*<0.001 and *F*_15,165_=25.6, *P*<0.001 for DA, DOPAC and HVA, respectively] (Fig. 3C, Fig. S6A,C). Similar to amphetamine, treatment with LY341495 caused an increase, although not as robust, in the extracellular DA levels in WT animals (factor time×genotype interaction: *F*_15,15_0=4.7, *P*<0.001) (Fig. 3F). Contrary to amphetamine effect, increase in extracellular DA was paralleled with the increased metabolic rates of DA (factor time×genotype interaction: *F*_15,150_=1.9, *P*<0.05 and *F*_15,150_=4.8, *P*<0.001 for DOPAC and HVA, respectively) (Fig. S6B,D). All the described neurochemical effects of amphetamine and LY341495 were absent in DAT-N157K mutant rats (Fig. 3C,F; Fig. S6).

## DISCUSSION

The subcortical dopaminergic system plays a key role in motivation, social processes and emotional responses (Berridge and Robinson, 1998; Schultz, 1998; Spanagel and Weiss, 1999; Klanker et al., 2013; Gunaydin et al., 2014). Alterations within this system are also critical for several psychiatric conditions. One molecular substrate that is essential for homeostasis and function of the subcortical dopaminergic system is DAT (Jones et al., 1998). On the genetic and molecular level, DAT is an essential element of the RDoC matrix. We therefore generated a novel genetic rat model with a loss of function of DAT in order to systematically study the consequences of innate imbalance of the dopaminergic system within the RDoC matrix. To induce a loss of function of the *Slc6a3* target gene that encodes for DAT, we performed an ENU-driven target-selected mutagenesis screen in rats (van Boxtel et al., 2011; Schneider et al., 2015). Point mutations in genes of interest can be rapidly introduced by the mutagen ENU (van Boxtel and Cuppen, 2011) and ENU mutagenesis can be combined with *a priori* target selection (van Boxtel et al., 2011). In this screen, we identified a functional point mutation in the *Slc6a3* gene. Our comprehensive *in silico* and molecular analysis of the DAT-N157K mutation revealed that although DAT is transcribed and translated in the mutant rat, it is not correctly processed to the cell surface. As a result, transport capacity is strongly reduced, extracellular DA levels are strongly augmented in subcortical areas and compensations in the 5-HT and NE systems occur. In cortical areas like the PFC, the dopaminergic system is not affected in DAT-N157K mutant rats. All this is characteristic for a hyperactive subcortical dopaminergic system and we further asked what would be the behavioral consequences within the different RDoC domains. Behavior in all five domains was affected by this mutation. Thus, mutant rats failed to show conditioned fear responses, were unable to learn stimulus-reward association, showed impaired cognition and social behavior, and were hyperactive. Our new treatment approach, the blockade of mGluR2/3 receptors by LY341495, could revert hyperactivity in DAT-N157K mutant rats. This treatment effect was very similar to that seen after amphetamine and atomoxetine treatment, which are standard medications to treat ADHD. We therefore propose DAT-N157K mutant rats as a model for further drug development targeting a hyperdopaminergic state as a specific disease mechanism.

The N157K point mutation in the *Slc6a3* target gene led to a subcortical hyperdopaminergic state. The DA system in the PFC was not affected by this mutation. This is not unexpected, since it is known that in the PFC DA clearance is mainly carried out by NET (Morón et al., 2002). From DAT-knockout mice, it is known that compensation mechanisms can occur in DA receptors and other monoaminergic systems (Gainetdinov and Caron, 2003). In order to monitor the compensatory changes in the brains of DAT mutant animals, we studied D1- and D2-like DA receptor binding, measured concentrations of monoamines and their metabolites in different brain sites, and examined changes in NET and SERT surface expression. D1 and D2 DA receptor binding was strongly downregulated in subcortical areas of mutant rats, while expression of the D1 receptor in the PFC was not changed. Intracellular DA levels in several subcortical brain areas of DAT-N157K mutant rats are very low when compared with the WT controls, whereas metabolic rate was increased, demonstrating an increased metabolic degradation of DA. Furthermore, we found that loss of DAT functionality was linked to diminished intracellular NE and 5-HT levels, as well as to reduced NET and increased SERT expression. No changes in NE and 5-HT levels or metabolic rates were detected in the PFC of mutant rats when compared with WT animals, confirming the insignificant role of DAT in this brain area.

Behavioral alterations in mutant animals may originate not only from a direct effect of DAT deficiency and dopaminergic imbalance, but also from the aforementioned compensatory neurochemical alterations. Behavioral performance of rats was investigated in all RDoC domains. Testing responses to aversive situations and contexts (domain negative valence system) in DAT mutant rats demonstrated the involvement of a subcortical hyperdopaminergic state in the expression of cue- and context-conditioned fear memory. Involvement of this system in fear conditioning was already demonstrated in earlier studies (Katoh et al., 1996; Martinez et al., 2008; Vucković et al., 2008). By contrast, behavior in a plus-maze test was not different between DAT mutant rats and their WT littermates. The RDoC matrix also does not distinguish DA as significant contributor for anxiety-related behavior.

Reduced sensitivity to reward and impaired social behavior was measured in DAT-N157K mutant rats. Both deficits are recognized as characteristic symptoms for diseases such as schizophrenia and mood disorders (Cannon et al., 1997; Russo and Nestler, 2013; Millan et al., 2014). However, DAT is not listed as a critical element in the domain of social processes. The reason for this is that none of the published results on DAT-knockout mice indicate an involvement of DAT in social behavior. However, since (1) in comparison to rats, mice are not a good model system for studying social behavior (Abbott, 2004), and (2) site-specific viral manipulation of DAT function in the Acb affected social behavior in rats (Adriani et al., 2010), and (3) activity dynamics of a VTA-Acb projection can encode and predict key features of social interaction (Gunaydin et al., 2014), we hypothesized and demonstrated that DAT-N157K mutant rats behave differently in a social interaction test. Our results suggest that the RDoC matrix should be updated with DAT and DA as critical elements for social behavior.

Cognition is crucial for the survival of all species, as it allows the organism to adapt to an ever-changing environment. Dopaminergic signaling plays a key role in cognitive control (Goschke and Bolte, 2014). Unbalanced dopaminergic activity, i.e. either excessive or insufficient, impairs cognitive performance and is a characteristic feature of several neurological and mental disorders, such as schizophrenia and ADHD (Cools and D'Esposito, 2011). Indeed, DAT mutants have impaired short-term memory and spatial learning in the novel-object recognition test and the spontaneous alternation test. This is in line with earlier experiments on striatal lesions that led to impaired spatial navigation in rats (Pistell et al., 2009), and experiments in DAT-knockout mice that showed poorer performance in the Y-maze test compared with WT mice (Li et al., 2010).

The most apparent effect in DAT-N157K mutants was a dramatically increased locomotor activity. This heightened activity is likely to be caused by a hyperactive extrapyramidal motor system, which has also been demonstrated for both DAT-knockout and -knockdown mice (Giros et al., 1996; Zhuang et al., 2001).

In the final set of experiments, we tested whether administration of amphetamine and atomoxetine, two clinically used medications for the treatment of ADHD (Wigal, 2009), would revert hyperactivity in mutant DAT-N157K rats. Indeed, both treatments reduced activity in mutant rats but had differing effects on WT animals – amphetamine had a strong psychostimulatory effect, whereas atomoxetine was sedative. Amphetamines are considered the most effective hyperactivity-reducing medications; however, the potential for cardiovascular and psychiatric problems limits their use (Wigal, 2009). Compared with amphetamine, the mGluR2/3 antagonist LY341495 only slightly increased extracellular DA output in WT rats. This increase did not have a psychostimulatory effect on the behavior of these animals. Nevertheless, this treatment reduced hyperactivity in mutant animals to the same extent as amphetamine or atomoxetine. The mechanism for such a reduction is not currently clear.

This is, to the best of our knowledge, the first systematic study of a disease mechanism, namely the occurrence of a wide range of neurochemical alterations, such as subcortical hyperdopaminergic state, caused by a conventional *Slc6a3*_N157K mutation, within the context of the RDoC approach. We not only validate and extend the role of DAT within the RDoC matrix, but we also propose mGluR2/3 antagonism as a new medication to target a hyperdopaminergic state. This disease mechanism may occur in patients suffering from schizophrenia (Laruelle et al., 1999; Weinstein et al., 2016), ADHD (Ohno, 2003), OCD (Pauls et al., 2014) and alcoholism (Tupala et al., 2001; Hirth et al., 2016). Although a diagnostic marker for a subcortical hyperdopaminergic state in those patients is yet to be developed, the use of positron emission tomography to study the dynamics of psychostimulant-induced dopamine release might offer such a diagnostic possibility (Mach et al., 1997).

Our study demonstrates that it is possible and advantageous to standardize animal research to the RDoC framework. Achieving the goal of neurobiologically driven categories in psychiatry, animal models with clearly defined genetic alterations along with well-characterized molecular adaptations will allow a more precise description of the pathophysiology and provide a reliable basis for development of RDoC-based treatments.

## MATERIALS AND METHODS

### Generation of *Slc6a3*_N157K mutant rats

Ten-week-old F344 male rats were injected intraperitoneally with N-ethyl-N-nitrosourea (ENU, 3×65 mg/kg, once weekly). Mutagenized males were then outcrossed with wild-type F344 females to produce the first generation (G1) of progeny which are heterozygous for a unique set of point mutations.

A total of about 3300 G1 rats were screened for mutations in a set of target genes with the *Slc6a3* gene being one of these target genes. Heteroduplex analysis by temperature gradient capillary electrophoresis was used as the method of choice for mutation detection.

A drawback of the ENU technology is the induction of bystander mutations that can contribute to the observed neurochemical and behavioral changes (Keays et al., 2006; Pawlak et al., 2008). In order to avoid the problem of bystander mutations *Slc6a3*_N157K founder rats were backcrossed with F344 female rats for up to 13 generations. Experiments were then done with 3- to 6-month-old male homozygous *Slc6a3*_N157K mutant rats (DAT-N157K) and their wild-type littermates (WT). All experimental procedures were approved by the Committee on Animal Care and Use, and carried out in accordance with the local Animal Welfare Act and the European Communities Council Directive of 24 November 1986 (86/609/EEC). See supplementary Materials and Methods for more information.

### Molecular mechanics and biochemical studies

Methods for molecular mechanics, generation and analysis of subconfluent HEK293 cells transiently transfected with rDAT-wild-type and rDAT-N157K, striatal DA uptake, brain microdialysis, receptor/transporter autoradiography, neurochemical analysis of tissue punches are provided in the supplementary Materials and Methods.

### Behavioral tests

All testing was carried out in a blind fashion with respect to genotype.

#### Cued and contextual fear conditioning

All training and testing procedures were performed in two different chambers (context A and B) located within a sound-attenuating cubicle (Coulbourn Instruments, Allentown, PA, USA). The apparatus was controlled by a personal computer equipped with FreezeFrame software (Actimetrics Software) and an IMAQ-A6822 interface card (National Instruments). Movements of the animal were recorded with a digital video camera mounted on the ceiling of the cubicle and analyzed using FreezeView software (Actimetrics Software).

Context A was used for acquisition of conditioned fear and for assessment of contextual freezing. Context A was a metal 17×18×32 cm box (H10-11M-TC, Coulbourn Instruments) with two opaque and two transparent walls. Stainless steel bars were used as a floor. During acquisition of conditioned fear, the scrambled electric footshock was delivered from a precision animal shocker (H13-15, Coulbourn Instruments) and the sound stimulus (cue) was generated with a conventional sound card, amplified with a HiFi amplifier (PR530A, Pyramid) and delivered via speakers in the chamber walls. Context B was used for cue recall and extinction. Context B was round black plastic box of 28 cm diameter and a flat, plastic tray floor. The sound stimulus was identical to that in context A.

On day 1, rats (*n*=5-6) were placed in context A and given five conditioning trials (with 3 min average inter-trial interval) consisting of cue presentations (30 s tone, 5 kHz, 77 dB) that co-terminated with footshocks (1 s, 0.5 mA). No further footshocks were given to animals for the remainder of the experiment. On day 2, rat was returned to the context A for 6 min to assess context-induced freezing, no auditory cues were presented. On day 3, a rat was placed in the context B for cue recall and extinction training: the session consisted of 2 min baseline activity measurement, 3 min cue presentation and thereafter another 15 random 30 s cue presentations. On day 4, the rat was tested for another cue recall session in context B, which started with 2 min of baseline activity measurements and 3 min of cue presentation. Context- and cue-induced freezing time was used for data analysis.

#### Elevated plus-maze (EPM)

The EPM consisted of a plus-shaped apparatus made of dark grey PVC elevated 50 cm above the floor with two opposing open arms (12×50×50 cm) which were illuminated at 80 lux and two enclosed arms (12×50×50 cm). All arms extended from a central square (10×10 cm). At the beginning of each trial, the rat (*n*=9-15) was placed in a closed arm of the EPM. Each rat was videotaped for 5 min and the following behaviors were analyzed: number of entries into open or closed arms (an entry was defined if all four paws were placed on that arm), time spent in open and closed arm(s), percentage of open arm entries [open arm entries/(open+closed arm entries)×100] and percentage of time spent in open arms [open arm time/(open+closed arm time)×100] were also calculated. The observation program ‘Viewer’ (Biobserve, Bonn, Germany) was used to analyze behavior.

#### Sucrose preference test

For sucrose preference test, rats (*n*=16-25) were separated into single cages and then given *ad libitum* access to one bottle with tap water and another bottle with 0.5% solution (w/v). The first 24 h of drinking were considered as a habituation phase. Thereafter, the positions of bottles were changed to avoid location preferences and subsequent 24 h intake of water and sucrose solution was measured. From these values, sucrose solution preference over water was calculated.

#### Autoshaping learning task

Animals (*n*=11-15) were weighed and food-restricted 1 day before the experiment started (i.e. food was removed from the food hoppers). In addition, 50 pellets (45 mg, Bio-Serv, Frenchtown, NJ, USA) were placed into the home-cage to avoid food neophobia. During the training days, the animals received 15 g of chow per rat in their home cage immediately after the session.

Autoshaping learning task was conducted in 12 identical standard operant conditioning chambers (31×27×33 cm, MED Associates, East Fairfield, VT) enclosed in sound- and light-attenuating cubicles (model ENV-022 V, Med-Associates) and connected to a computer through an interface and controlled by MED-PC software. Each chamber was equipped with a white house light centered 19 cm above two response levers (model ENV-112CM; positioned 7 cm above the floor), sound generator (model ENV-223AM) and a food dispenser (model ENV-203), which delivered food pellets (45 mg, Formula A, Noyes precision pellets, Research Diets, New Brunswick, NJ, USA) on one side. Two stimulus lights were positioned above each lever, and another light illuminated the food tray. The nose-poke operandum (model ENV-114 M) was situated on the opposite side of the chamber.

For food-magazine training, each rat was placed in an individual experimental chamber for a habituation period and had access to 50 food pellets previously placed inside the food magazine. Thirty minutes later, rats were removed from the boxes and remaining pellets counted.

The autoshaping training consisted of discrete trials. During each trial, one retractable lever was extended into the chamber (reward-predicting stimulus). Eight seconds later or after a lever-press response (whichever came first), the lever was retracted and a single Noyes precision pellet was delivered followed by a variable inter-trial interval with the average of 60 s (30-90 s). On three consecutive days the training sessions consisted of 20 trials and on the fourth day the test session consisted of 40 trials. At the end of each session, the remaining pellets were counted. The number of responses (i.e. pellets earned) was recorded.

#### Novel object recognition test

The recognition test was performed in the open-field apparatus (see below). All rats (*n*=22-23) were habituated to the test room and the open field 1 day prior to the test. The objects to be discriminated were made of glass and existed in multiple copies. All objects were cleaned with 50% ethanol and distilled water and thoroughly dried before and between testing phases. Preliminary testing of all objects chosen for this test indicated an equal attractiveness to the animals.

The test consisted of an initial 3 min sample phase (P1) and a 3 min discrimination phase (P2), which were separated by an inter-trial interval of 15 min. During P1, the rat was placed in the center of the open field and exposed to two identical unknown objects (A), then the rat was returned to the home cage. The rat was placed back in the open field after 15 min for object discrimination in P2 and now exposed to the familiar object (A′, an identical copy of the object presented in P1) and a novel test object (B). Exploration of the objects (sniffing, touching with whiskers, and licking) was recorded during P1 and P2. Sitting beside or standing on top of the objects was not scored as object investigation. For the calculation of object discrimination the exploration time of the novel object was expressed as percentage of the total exploration time of both objects during P2 [100/(A′+B)×B].

#### T-maze spontaneous alternation test

To test the animals’ working memory, their spontaneous alternation behavior was examined using a T-maze made of dark PVC (70×70×113 cm arm length) and a small start compartment adjacent to the long arm. During the habituation, animals (*n*=5 per genotype) were allowed to explore the test apparatus for 3 min during two consecutive days prior to the test. Each session of the T-maze test consisted of two trials: the sample trial and the choice trial. The test period started by placing the rat into the start area for 20 s before the guillotine door was opened. During the sample trial, one of the arms (goal arm) was blocked (in an alternating way). Therefore, the animal could only enter and freely explore the open arm (sample arm). As soon as the rat had entered the arm entirely (including the tail), the door behind it was shut for 20 s, then the rat was put back into the start area. Within an inter-trial interval of 60 s maximum, the apparatus was cleaned with 30% ethanol. Thereafter, the rat had to pass the choice trial where both arms were freely available. The choice was accredited as a ‘correct choice’ when the animal entered the goal arm. If the test subject revisited the already explored sample arm, the choice was marked as ‘wrong’.

#### Social interaction test

Social interaction with an unfamiliar social partner (7- to 8-week-old male Fischer rat) was performed in an open field (see below). All rats (*n*=11-12) were habituated to the test room and the open field 1 day prior to the test. For the test, each rat was placed in the center of the box for 1 min prior to placement of an unfamiliar social partner. Social interaction was then assessed during the subsequent 5 min. The following behavioral elements were quantified only for the test rat: (1) social behavior, including contact behavior (grooming and crawling over), social exploration (anogenital and non-anogenital investigation) and approach/following; (2) social evasion was scored as an active withdrawal from social contact; and (3) self-grooming behavior as stress- and anxiety-related behavior.

### Home-cage activity

Home-cage activity was monitored by the use of an infrared sensor connected to the recording and data storing system Mouse-E-Motion (Infra-e-motion, Henstedt-Ulzburg, Germany). Rats were placed in single cages (*n*=9-11) and a Mouse-E-Motion device was placed above each cage (30 cm from the bottom) so that the rat could be detected at any position inside the cage. The device sampled every second whether the rat was moving or not. The sensor could detect body movement of the rat of at least 1.5 cm from one sample point to the successive one. The data measured by each Mouse-E-Motion device were downloaded into a personal computer and processed with Microsoft Excel.

### Open-field test

To measure the locomotor activity in a novel environment, an open-field apparatus was used. The open field was a dark PVC box of four equal squares (51×51×50 cm). The light was adjusted to 50 lux (measured in the center of a square). To measure rearings, each box contained two opposing sensor barriers about 15 cm above ground level. A center zone (24.9×22.2 cm) was defined in the middle of each box. Distance traveled, center time and the number of rearings were recorded digitally for a period of 60 min. All animals were habituated to the experimental room 1 day prior to the test. For the test, the animal was placed in the center of the box (*n*=20-24) and the experimenter then left the room while a camera above the apparatus recorded the animal's movements. The observation program ‘Viewer’ (Biobserve, Bonn, Germany) was used to analyze the behavior.

### Pharmacological studies

Full details of neurochemical and pharmacological studies can be found in supplementary Materials and Methods.

### Statistical analysis

The data derived from fear-conditioning, EPM, sucrose preference, novel object recognition, T-maze, social interaction (i.e., anogenital exploration, non-anogenital exploration, approach/following behavior, total interaction events, exploration time, self-grooming and social evasion) and open field activity (i.e., total distance, number of rearings and center time) tests were analyzed by independent two-tailed *t*-test. Two-way ANOVA with repeated measures was used for analysis of autoshaping leaning task (factors: genotype and session), home-cage activity measurements and the distance traveled every 10 min in the open field test (factors: genotype and time). It was also used for analysis of drug treatment effect on locomotor activity (factors: treatment group and time) and analysis of extracellular neurotransmitter levels (factors: genotype and time). One-way ANOVA was used for analysis of drug treatment effect on total distance, number of rearings and center time in the open-field activity tests (factor: treatment group). Whenever significant differences were found, *post hoc* Student Newman–Keul's tests were performed. The chosen level of significance was *P*<0.05.
